# Analysis of promoters and CREB/AP-1 binding sites of the human TMEM174 gene

**DOI:** 10.3892/etm.2013.1275

**Published:** 2013-08-28

**Authors:** FEN HU, YANAN MENG, LIXIA GOU, XIUJUN ZHANG

**Affiliations:** 1 College of Life Sciences, Hebei United University, Tangshan, Hebei 063000;; 2 Department of Clinical Laboratory, Baoding People’s Hospital, Baoding, Hebei 071051, P.R. China

**Keywords:** activator protein-1, core promoter, cyclic-AMP response element binding protein, transmembrane protein 174 gene, transcriptional regulation, luciferase

## Abstract

Transmembrane protein 174 (TMEM174) is a type III transmembrane protein with no clear signal peptide. The N and C terminals are located inside the cell. Our previous study demonstrated high expression of TMEM174 in the kidney and its potential involvement in renal cancer based on its capacity to stimulate cell proliferation. However, the mechanism by which TMEM174 promotes proliferation at the transcriptional level remains to be elucidated. In the present study, the TMEM174 promoter region was amplified from whole blood DNA. Six different regions of the regulatory sequences of the TMEM174 promoter region including ~2.5 kb of the upstream region were cloned into the dual luciferase expression vector pGL3-basic. Comparison of the activity of these fragments in dual luciferase reporter assays revealed higher levels of activity for the fragments spanning −186 to +674, −700 to +674, −1,000 to +674 and −2,500 to +1 bp. Lower levels of activity were detected for the fragments spanning −466 to +674 and −890 to +674 bp. The highest activity was detected for the fragment spanning −186 to +674 bp. Electrophoretic mobility shift assay (EMSA) was performed to determine effective transcription factor binding sites. Specific binding of the cyclic-AMP response element binding (CREB) within the TMEM174 gene promoter region was demonstrated, although binding of the activator protein-1 (AP-1) was also detected in this region. In conclusion, these results suggest that the core promoter region of the human TMEM174 gene is located within the region spanning −186 to +674 bp and that the transcription factors CREB and AP-1 are involved in the transcriptional regulation of this gene.

## Introduction

Transmembrane protein 174 (TMEM174) was identified among a large pool of genes by high-throughput cell screening technology, which is used to isolate functional genes and to provide insight into the mechanisms of gene function (1). Genes are investigated using a reverse biological strategy in which gene expression profiling of tissues and cell lines is performed. This approach revealed high expression levels of TMEM174 in kidney tissue and the lymphadenoma-derived Raji cell line (2). The TMEM174 gene is activated by the activator protein-1 (AP-1) pathway in HeLa and 293T cells (~3- and 9-fold, respectively). Preliminary studies of the mechanism underlying the promotion of cell proliferation indicated that TMEM174 is linked with Ras and Raf in the extracellular-signal-regulated kinase (ERK) pathway. Furthermore, TMEM174 has a role in promoting the G2/S phase of the cell cycle (2). However, the underlying mechanism remains to be elucidated and other pathways and transcription factors have been suggested to be involved in this process.

The cyclic-AMP response element binding (CREB) protein is a transcription factor that affects a spectrum of cellular activities. including glucose homeostasis, growth factor-dependent survival, proliferation, differentiation and memory (3,4). CREB recruitment to the cyclin D1 promoter promotes cyclin D1 transcription and cell proliferation (5). Transgenic animal studies have shown that overexpression of CREB confers oncogenic characteristics on cells in various tissues and that abnormal CREB expression is associated with tumor development in humans (6). CREB plays an important role in the development of brain tumors, leukemias and other types of cancer (7). Evidence based on the high level of homology of zebrafish CREB and its mammalian counterpart suggests that activated (phosphorylated) CREB is localized in proliferation zones (7). Activation of CREB has been suggested to stimulate cellular proliferation through the PI3-K/AKT pathway (8) and to act as a crucial transcription factor for the regulation of TMEM174 expression.

AP-1 family members are important upstream activators of the ERK signaling pathway (9), which are involved in cell proliferation, transformation, differentiation and death (10–12). AP-1 is a collective term referring to dimeric transcription factors composed of Jun, Fos, activating transcription factor (ATF) and musculoaponeurotic fibrosarcoma (MAF) protein subunits that bind to a common AP-1 binding site (13,14). c-Jun is a positive regulator of cell proliferation, while JunB mediates the converse effect. Jun promotes apoptosis by participating in cell stress-induced transcriptional activation of apoptotic target genes (15). Previous studies have indicated that TMEM174 overexpression activates AP-1, since the upstream molecules ERK, ELK-1 and Fos are markedly activated or stimulated by sequential blockade of these upstream factors in the ERK pathway (2).

These studies contribute to the understanding of the mechanism by which cell proliferation is promoted at the protein level. However, details of the mechanism at the transcriptional level remain to be elucidated. Identification of the core promoter is a critical first step in this process, facilitating subsequent determination of the important transcription factors.

In the present study, the mechanism by which TMEM174 promotes cell proliferation at the transcriptional level was investigated. The promoter region and transcription factor binding sites were predicted by bioinformatics analysis. Among the large numbers of binding sites predicted, such as CREB, AP-1, nuclear factor-κB (NF-κB) and Oct1, CREB and AP-1 were identified with potential binding sites for interaction with the TMEM174 promoter region by electrophoretic mobility shift assay (EMSA).

## Materials and methods

### PCR amplification and molecular cloning

Primers were designed for the amplification of fragments of various lengths corresponding to the 4.8 kb sequence upstream of the TMEM174 gene (available in the NCBI mapview database: http://www.ncbi.nlm.nih.gov/mapview) and using TFEARCH online software (http://www.cbrc.jp/research/db/TFSEARCH.html) to predict the candidate promoter region and transcription factor binding sites. Fragments were amplified for cloning from whole blood genomic DNA. The primers used are shown in Table I.

The PCR amplification reaction system (Table II) conditions were as follows: initial denaturation at 95°C for 5 min; 30 cycles of 95°C for 30 sec; 55–60°C (depending on the primer pair used) for 30 sec and 72°C for 1 min and final elongation at 72°C for 7 min. The PCR products were purified by agarose gel electrophoresis (Sigma-Aldrich, Schnelldorf, Germany) and Vigorous purification kits (Vigorous Biotechnology, Beijng, China). The purified products were cloned into the *Kpn*I and *Hin*dIII restriction enzyme sites of pGL3-basic (Promega, Madison, WI, USA). The recombined vectors were sequenced by SinoGenoMax Co., Ltd. (Beijing, China).

### Transfection and dual luciferase reporter assay

293T cells were seeded in 96-well plates (1×10^4^/well) in complete Dulbecco’s modified Eagle’s medium (DMEM; HyClone, Logan, UT, USA) containing 10% fetal bovine serum (FBS) and were transfected the next day with Vigofect (Vigorous Biotechnology) according to the instructions provided by the manufacturer. A total of 104 ng plasmid DNA/well was transfected, including 100 ng recombinant pGL3 plasmid or pGL3-basic (empty vector control) and 4 ng pRL-TK (Promega) containing the *Renilla* luciferase gene as an internal control. Transfections were performed in triplicate. The cells were lysed in standard 1X lysis buffer (Promega) for 30 min at room temperature (RT) and the cell lysates were assayed for both firefly and *Renilla* luciferase activity using the Dual-Luciferase Reporter assay kit (Promega) according to the instructions provided by the manufacturer. Fluorescence was detected using a GENius Pro microtiter plate reader (Tecan, Männedorf, Switzerland). Relative luciferase activity was determined by normalizing the activity of the firefly luciferase activity (F value) against the *Renilla* luciferase activity (R value). Each experiment was performed at least three times. The F/R value was calculated respectively and an average value was obtained as the recombined plasmid activity. pGL3-basic was used as the negative control and its activity was defined as 1. The activities of all other recombined plasmids were compared with the negative control.

### EMSA

Nuclear extracts of HepG2 cells were prepared using Nuclear Extract kits (Active Motif, Carlsbad, CA, USA). Protein concentrations were determined using BCA protein assay kits (Vigorous Biotechnology). The oligonucleotide probes (CREB, AP-1 and competitive probes) used in EMSA were designed according to a computer-based search with the software Promoter Scan (ProScan version 1.7 suite of programs developed by Dr Dan Prestridge, http://www-bimas.cit.nih.gov/molbio/proscan/). Experimental groups were as follows: CREB probe/nuclear extract, AP-1 probe/nuclear extracts, CREB probe/nuclear extract + competitive probe and AP-1 probe/nuclear extract + competitive probe. Free probe was used as a negative control. Nuclear extracts (5 *μ*g) were incubated with 2 *μ*l (200 nM) biotin-labeled DNA probe and mixed with 10X binding buffer (2 *μ*l), 50% glycerol (1 *μ*l), MgCl_2_ (1 *μ*l), Poly (dI.dC) (1 *μ*l) and 1% NP-40 (1 *μ*l) for 10 min at RT. Bound DNA complexes were separated by non-denaturing polyacrylamide gel (6.5%) electrophoresis and transferred to a positively charged nylon membrane. Cross-linking was performed under ultraviolet (UV) light for 20 min and the membranes were probed with a streptavidin-horseradish peroxidase (HRP) conjugate and incubated with the chemiluminescent substrate for band detection. For competition experiments, unlabeled competitor oligonucleotides were pre-incubated (200-fold excess) with the labeled probe.

### Statistical analysis

Differences between the levels of expression were analyzed by one-way ANOVA. P<0.05 was considered to indicate a statistically significant difference.

## Results

### TMEM174 promoter cloning

**Figure 1.**
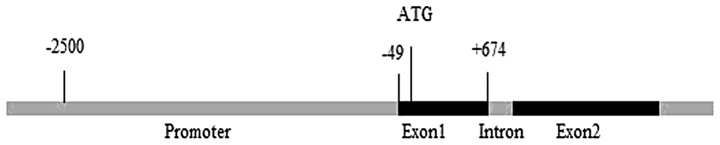

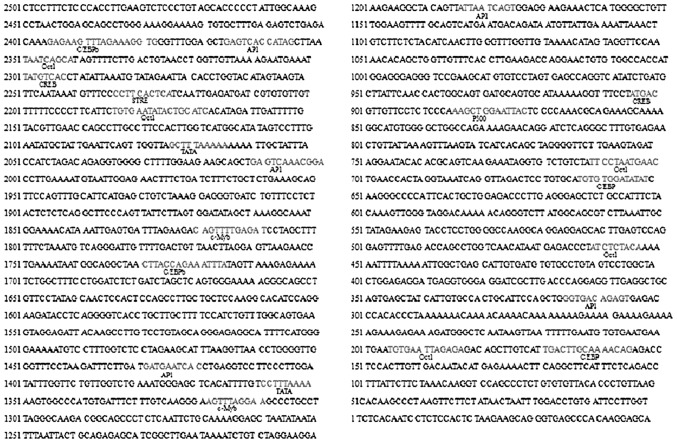
(A) Promoter region of the TMEM174 gene according to the Ensembl genome database. TMEM174 consists of 732 bp encoding putative 243 amino acids containing 2 exons. In the present study, the promoter region and the first exon were cloned into pGL3-basic. (B) Sequence of part of the promoter region showing potential transcription factor binding sites predicted by TFSEARCH (C/EBPb, AP-1, Oct1, CREB, STRE, c-Myb, P300). Selected transcription factors are shown. Nucleotides are numbered from the start codon ATG. AP-1, activator protein-1; CREB; cyclic-AMP response element binding.

The promoter region of TMEM174 was amplified from whole blood genomic DNA as described in Materials and methods. Five fragments of various lengths corresponding to the predicted promoter region and the first exon of TMEM174 were amplified: −186 to +674, −466 to +674, −700 to +674, −890 to +674 and −1,000 to +674 bp. Amplified products of 860, 1,140, 1,347, 1,564 and 1,674 bp were cloned into pGL3-basic. The cloned sequences were identical to the original genomic sequences shown in Fig. 1A. Numerous transcription factor binding sites were predicted within the promoter region by TFSEARCH, including CREB, AP-1, P300, NF-κB and Oct1 (Fig. 1B).

### Characterization of TMEM174 promoter activity

**Figure 2.**
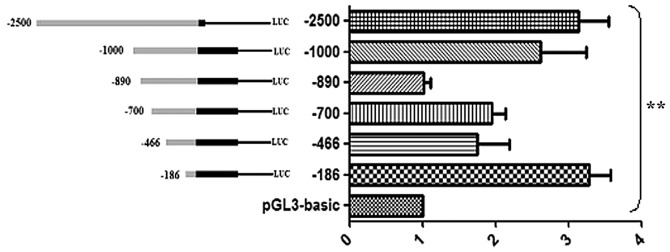
Dual luciferase reporter gene assays of TMEM174 gene promoter constructs. Various recombinant vectors and pRL-TK were co-transfected into 293T cells. pRL-TK and pGL3-basic were used as internal and negative controls, respectively. Relative luciferase activity was determined by normalizing the activity of firefly luciferase against *Renilla* luciferase activity. Data represent the mean ± SD of three independent experiments. ^**^P<0.05. The P-value was calculated using one-way ANOVA that compared the activities of the 7 plasmids.

Dual luciferase reporter assays were performed to detect promoter activity. Analysis of the recombined plasmids following transfection into 293T cells indicated that fragments −186 to +674, −700 to +674, −1,000 to +674 bp and −2,500 to +1 bp exhibited higher levels of activity compared with fragments −466 to +674 and −890 to +674 bp, with the two highest levels of activity detected for fragments −186 to +674 and −2,500 to +1 bp. Significant differences between groups are indicated in Fig. 2.

### Identification of transcription factors binding to the human TMEM174 promoter region

**Figure 3.**
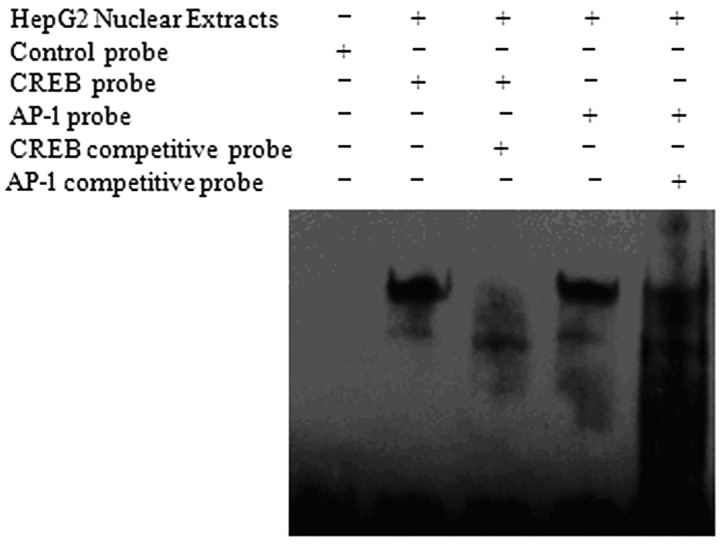
Binding activity of CREB and AP-1. From left to right, samples are control probe, CREB probe/nuclear extract, AP-1 probe/nuclear extract, CREB probe/nuclear extracts + competitive probe and AP-1 probe/nuclear extract + competitive probe. CREB and AP-1 bind with the promoter region. CREB binding was eliminated in the presence of the competitive probe, while AP-1 binding was partially blocked. CREB; cyclic-AMP response element binding; AP-1, activator protein-1.

The identification of transcription factor binding sites within the predicted promoter region is important in determining the mechanism of transcriptional activation of this gene. Numerous transcription factor binding sites were identified within the predicted promoter region. Among these, CREB and AP-1 were related to cell proliferation. EMSA was performed as described in Materials and methods to investigate transcription factor binding. These experiments demonstrated specific binding of CREB with the TMEM174 promoter. The AP-1 probe designed according to the predicted TMEM174 promoter AP-1 binding site bound to HepG2 nuclear extract; this binding was partially inhibited by the addition of a competitive probe, indicating that AP-1 binds non-specifically within the TMEM174 promoter region (Fig. 3).

## Discussion

A previous study focused on the mechanism by which TMEM174 promotes cell proliferation at the protein level have indicated potential signal transduction pathways involved in this process (2). The present study aimed to further elucidate this mechanism at the transcriptional level. Investigations of the activity of the predicted promoter showed that fragments −186 to +674, −700 to +674, −1,000 to +674 and −2,500 to +1 bp exhibited higher levels of activity compared with fragments −466 to +674 and −890 to +674 bp. The fragment −186 to +674 bp exhibited the highest activity indicating that the core promoter region might be located within this region of the TMEM174 promoter. Furthermore, the variation detected in the levels of activity demonstrated the regulatory complexity of this promoter. Based on the results of these experiments, the regions spanning −186 to −466 and −700 to −890 bp are suggested to contain strong negative regulatory elements. These data indicate that the apparent changes in promoter activity are the result of complex interactions between different regulatory elements.

TMEM174 has been identified as a potential regulator of cell proliferation using high-throughput cell screening technology (data not shown). This method allows the rapid identification of functional genes from a large pool and provides insights into to the function of these genes. However, this method is limited in its application for subsequent studies of the mechanism of gene function due to the potential omission of the role of such proteins in other pathways.

RNA *in situ* hybridization analysis showed that TMEM174 is highly expressed in some types of renal cancer, such as squamous cell carcinoma with necrosis, papillary renal cell carcinoma and transitional cell carcinoma, and that TMEM174 is expressed in the majority of renal cancers and pyelonephritis (data not shown). These data indicate that TMEM174 plays a role in the development of renal cancer.

NF-κB, STAT3, AP-1, CREB and nuclear factor erythroid 2-related factor (Nrf2) are transcription factors that regulate tumor cell proliferation, transformation, survival, invasion, angiogenesis, metastasis, chemoresistance and radioresistance (16). In the current study, sequence analysis identified numerous transcription factor binding sites within the predicted promoter sequence of TMEM174. Among these, CREB and AP-1 are involved in cell proliferation. EMSA indicated specific binding of CREB within the TMEM174 gene promoter region, while binding of the AP-1 was shown to be non-specific. Thus, CREB is suggested to be an important transcription factor involved in the regulation of TMEM174 gene expression, and the inhibition of CREB, and possibly AP-1, is suggested to inhibit the migration and invasion of cancer cells (17). Furthermore, cell line expression profiling showed that TMEM174 was expressed in the lymphadenoma-derived Raji cell line, indicating a role for TMEM174 in the development of lymphadenoma. CREB is overexpressed in the bone marrow of the majority of patients with acute myeloid leukemia (AML) and is associated with a poor initial outcome of clinical disease in AML patients. Moreover, CREB plays an important role in hematopoiesis (18–20). Thus, TMEM174 is suggested to play a role in leukemogenesis by activating CREB. Among the numerous transcription factors identified in the promoter sequence, CEBP has also been shown to be involved in inflammation (21). Therefore, TMEM174 is suggested to be involved in the regulation of pyelonephritis via CREB. However, these hypotheses require further investigation.

In conclusion, the results of the present study indicate that the core promoter region of the human TMEM174 gene is located in the region spanning −186 to +674 bp and that the transcription factors CREB and AP-1 are involved in the transcriptional regulation of this gene. These findings provide further insight into the mechanism of TMEM174 gene regulation, with the identification of CREB representing a basis for further studies concerning this mechanism.

## Figures and Tables

**Table I.** Primer sequences.

Primer	Primer sequences	Position (bp)
P1	F: 5′-CGGGGTACCGTTTTGCAAGTCAATGACAAGCTGTCTC-3′	−186 to +674
	R: 5′-CCCAAGCTTCTTTCACGGACGGTGGAAATCACAG-3′	
P2	F: 5′-CGGGGTACCCAGCCAATTTTTAAAATTTTTTGTAGAGATAGG-3′	−466 to +674
	R: 5′-CCCAAGCTTCTTTCACGGACGGTGGAAATCACAG-3′	
P3	F: 5′-CGGGGTACCCAGGAGTCTAACCTGATTTACCTAGTGGTTC-3′	−700 to +674
	R: 5′-CCCAAGCTTCTTTCACGGACGGTGGAAATCACAG-3′	
P4	F: 5′-CGGGGTACCGTTTGGGGAGTAATTCCAGCTTTGGG-3′	−890 to +674
	R: 5′-CCCAAGCTTCTTTCACGGACGGTGGAAATCACAG-3′	
P5	F: 5′-CGGGGTACCGACACATGCTTCGGACCCTCCCTC-3′	−1000 to +674
	R: 5′-CCCAAGCTTCTTTCACGGACGGTGGAAATCACAG-3′	
P6	F: 5′-CGGGGTACCACAGGGAGACTTCAAGGTGGGAGAAAGGAG-3′	−2500 to +1
	R: 5′-CCCAAGCTTCTTCTATAACTAATTTGGACCTGTGATTCCTTG-3′	

Restriction enzyme sites were *Kpn*I and *Hin*dIII. F, forward; R, reverse. Underlining indicates restriction enzyme sites.

**Table II.** PCR amplification reaction system.

Substance	Value (*μ*l)
2.5 mM dNTP mixture	2
10X Pyrobest™ buffer	2.5
Template DNA	0.1
Forward primer	2.5
Reverse primer	2.5
Pyrobest™ DNA polymerase	0.3
ddH_2_O	15.1
Total	25
